# Drug delivery, biomaterials and nanomedicine: an interview with Daniel S Kohane

**DOI:** 10.4155/fsoa-2016-0050

**Published:** 2016-08-19

**Authors:** Daniel S Kohane

**Affiliations:** 1 Laboratory for Biomaterials & Drug Delivery, Department of Anesthesiology, Division of Critical Care Medicine, Boston Children’s Hospital, Harvard Medical School, 300 Longwood Avenue, Boston, MA 02115, USA

**Keywords:** biomaterial, drug delivery, nanoparticle, nanoscience, stimulus-responsive

## Abstract

**Daniel Kohane speaks to Francesca Lake, Managing Editor:** Obtaining his MD and PhD in Physiology from Boston University (MA, USA), Dan Kohane went on to complete residencies in Pediatrics at Boston Children’s Hospital (MA, USA) and Anesthesiology at Massachusetts General Hospital (USA), followed by a fellowship in Pediatric Critical Care at Boston Children’s Hospital. He is currently a Professor of Anesthesia at Harvard Medical School (MA, USA) and a Senior Associate in Pediatric Critical Care at Boston Children’s Hospital, where he directs the Laboratory for Biomaterials and Drug Delivery. His research focuses on drug delivery and biomaterials, and in particular, nanomedicine.

## Q Can you tell us a little more about your current position & what led you to where you are today?

There are two components to my current position. I spend most of my time in my laboratory, which is populated with a combination of physical scientists interested in biomedical problems and physicians interested in engineering. We are focused on biomaterials, drug delivery and nanoscience. My other job is as a pediatric intensive care physician. Clinical duty takes me away from the laboratory, but I find it very rewarding.

The path to my current position involved a slightly ridiculously prolonged clinical training, but I returned to science in the end. I had the great good luck to begin that return in the laboratory of Professor Robert Langer at The Massachusetts Institute of Technology, where I was exposed to a broad range of areas of study and was given the freedom to pursue them. Eventually, I was fortunate to land my current position in the Department of Anesthesiology at Children’s Hospital Boston, which provides me an excellent setting for clinical work and is very supportive of my research.

## Q Much of your work lies in developing drug delivery systems. What are you working on at the moment in this arena?

We have many projects going: a drug-eluting contact lens for glaucoma and many other conditions (with Dr Joseph Ciolino at the Massachusetts Eye and Ear Infirmary), a method of treating middle ear infections by getting drugs to cross the eardrum, methods of targeting drugs to the retina, polymers to improve release profiles of drugs for glaucoma and other disease, triggerable drug delivery systems responsive to a range of stimuli and many others.

## Q What impact do you hope these will have in the clinic?

We hope that all of these inventions will be useful to patients. Some, such as the treatment for ear infections, will improve compliance and prevent systemic side effects and the development of antibiotic-resistant bacteria. Others, such as a treatment for sepsis, may save lives.

## Q When designing externally triggered & targeted drug delivery systems, what considerations need to be taken into account?

There are many considerations, which have been reviewed by others and us. They can include the drug, the vehicle, the anatomic location, the disease, the external trigger, the operator, the regulatory system, the intellectual property, the nature of clinical practice for the particular disease, the medical reimbursement system and many other factors.

## Q What are your top pieces of advice for others facing those considerations?

For people early in their careers, I would advocate cross-training. For people at all levels, I would strongly suggest seeking reliable advice. The difficulty with such advice is that many inventions seem like a good idea at first blush, but do not stand up to more exacting scrutiny. Good advice is hard to find.

## Q Your work also takes in pediatrics: what difficulties would be expected when translating such systems to pediatric populations?

The problems I am best acquainted have to do with guaranteeing protection to children during clinical trials. It may be necessary to do trials in adults first.

## Q What do you think the future looks like for drug delivery?

I think that drug delivery technology has the potential to greatly increase the safety and efficacy of many medications, and to give patients greater control over their medication regimens. There is also considerable interest in systems where implanted sensors act through closed feedback loops to adjust drug release from a depot.

## Q Your laboratory also takes in wider biomaterials work. What else are you currently working on?

We have a program for treating septic patients by withdrawing cytokines from the blood; our preliminary data suggest that it works in rodents. We are also very interested in developing surgical glues that are sufficiently biocompatible to use within the body; this is not easy. Other interests include the interface of nanoscience and tissue engineering, and surfaces that are resistant to fouling and biofilm.

## Q What do you enjoy the most about your work?

It is wonderful to watch bright young people from diverse academic backgrounds encounter fields different to their own and create exciting new things.

## Q And what do you find the most challenging?

I am frequently puzzled by grant reviewers who seem not to have actually read the proposal.

## Q Finally, if you had unlimited resources available, what would you do with them?

I would create a fellowship-based center where students from different disciplines could learn and create in this field. Participants would graduate with an enhanced understanding of the interaction between the basic scientific and biomedical considerations that underly progress in this very applied field. With truly unlimited resources, it would be possible to gather bright candidates irrespective of the resources of their country of origin. Over time, the hope would be that such a center would accelerate applied biomedical research across the globe. As it turns out, however, I do not have unlimited resources, so I am restricted to cultivating my own garden [1].
